# Ethical issues in direct-to-consumer healthcare: A scoping review

**DOI:** 10.1371/journal.pdig.0000452

**Published:** 2024-02-13

**Authors:** Ashwini Nagappan, Louiza Kalokairinou, Anna Wexler

**Affiliations:** 1 Department of Health Policy and Management, University of California, Los Angeles, Los Angeles, California, United States of America; 2 Department of Medical Ethics and Health Policy, University of Pennsylvania, Philadelphia, Pennsylvania, United States of America; 3 Center for Medical Ethics and Health Policy, Baylor College of Medicine, Houston, Texas, United States of America; Iran University of Medical Sciences, IRAN (ISLAMIC REPUBLIC OF)

## Abstract

An increasing number of health products and services are being offered on a direct-to-consumer (DTC) basis. To date, however, scholarship on DTC healthcare products and services has largely proceeded in a domain-specific fashion, with discussions of relevant ethical challenges occurring within specific medical specialties. The present study therefore aimed to provide a scoping review of ethical issues raised in the academic literature across types of DTC healthcare products and services. A systematic search for relevant publications between 2011–2021 was conducted on PubMed and Google Scholar using iteratively developed search terms. The final sample included 86 publications that discussed ethical issues related to DTC healthcare products and services. All publications were coded for ethical issues mentioned, primary DTC product or service discussed, type of study, year of publication, and geographical context. We found that the types of DTC healthcare products and services mentioned in our sample spanned six categories: neurotechnology (34%), testing (20%), in-person services (17%), digital health tools (14%), telemedicine (13%), and physical interventions (2%). Ethical arguments in favor of DTC healthcare included improved access (e.g., financial, geographical; 31%), increased autonomy (29%), and enhanced convenience (16%). Commonly raised ethical concerns included insufficient regulation (72%), questionable efficacy and quality (70%), safety and physical harms (66%), misleading advertising claims (56%), and privacy (34%). Other frequently occurring ethical concerns pertained to financial costs, targeting vulnerable groups, informed consent, and potential burdens on healthcare providers, the healthcare system, and society. Our findings offer insights into the cross-cutting ethical issues associated with DTC healthcare and underscore the need for increased interdisciplinary communication to address the challenges they raise.

## Author summary

Health products and services are increasingly being sold directly to consumers (DTC). However, prior research in this area has remained limited to specific medical domains, and there has not been a comprehensive examination of ethical concerns. To address this gap, we conducted a review of ethical concerns raised in the academic literature across various types of DTC healthcare products and services. Our search on PubMed and Google Scholar for publications discussing the ethics of DTC healthcare yielded 86 publications that met our inclusion criteria. Articles centered on six main categories of DTC products and services: neurotechnology, testing, in-person services, digital health tools, telemedicine, and physical interventions. Arguments supporting DTC healthcare included that it could promote increased access, autonomy, and convenience. The most frequently highlighted concerns centered on insufficient regulation, unproven efficacy, safety risks, misleading marketing claims, and privacy issues. Other frequently occurring ethical concerns revolved around financial costs, targeting vulnerable groups, informed consent, and potential burdens on healthcare providers, the healthcare system, and society. Overall, enhanced communication across medical specialties may be beneficial for navigating the ethical challenges raised by DTC healthcare.

## Introduction

An increasing number of healthcare products and services are being offered to the public on a direct-to-consumer (DTC) basis. While there is no single definition of what constitutes a DTC healthcare product or service, it typically involves consumers initiating the process of ordering a product or service, often online, with little to no direct involvement from a healthcare professional [1]. Examples include consumer neurostimulation devices [2], digital mental health apps [3], and laboratory tests [4]. Many, but not all, DTC health products are reliant on digital health platforms for their business model or product functionality.

Compared to the traditional model of healthcare delivery, DTC healthcare confers benefits to consumers such as increased convenience, access, and cost transparency [5–7]. However, DTC healthcare also raises ethical concerns that pertain to a diverse array of stakeholders, including consumers who utilize DTC products and services, physicians both within and outside DTC companies, and the healthcare system as a whole. Ethical concerns include those that are commonly recognized in medical ethics, such as the adequacy of informed consent and potential harms, as well as others related to the validity of tests, data privacy, and accountability [8–10].

To date, scholarship on DTC healthcare products and services has largely proceeded in a domain-specific fashion, with discussions of relevant ethical challenges occurring within specific medical specialties, such as genetics [10–12], audiology [13], neurology [14], psychiatry [15], orthodontics [16], and cardiology [17]. For example, geneticists have debated what constitutes appropriate communication of DTC genetic testing results to patients [10], neurologists have questioned the privacy of brain data from DTC neurotechnology headsets [14], and dentists have argued that DTC orthodontic services can harm consumers [16]. Other work has reviewed classes of products and services, such as DTC telemedicine [6], DTC prescription services [5], and DTC digital health [18].

There has been a marked lack of scholarship evaluating the cross-cutting concerns raised by DTC healthcare. Indeed, as one of us has previously argued, conceptualizing DTC products and services as a part of a larger phenomenon may yield valuable insights regarding convergent challenges [19]. Moreover, a comprehensive review through a bioethics lens is essential for providing a structured overview of the ethical concerns that span various medical specialties [20], for obtaining a nuanced understanding of the ethical complexities of DTC healthcare, and for establishing a foundation for well-informed policy. Thus, the objective of the present study was to conduct a scoping review of ethical issues raised in the academic literature across various DTC healthcare products and services. Specifically, we sought to characterize the types of DTC products and services examined by scholars and map the concordant ethical issues raised in the literature. In doing so, we aimed to generate a broader understanding of the common challenges arising from the societal shift toward DTC healthcare.

## Methods

Our scoping review of the literature on the ethics of DTC healthcare followed the methodology set out in Arksey and O’Malley and adhered to the Preferred Reporting Items for Systematic Reviews and Meta-Analyses extension for Scoping Reviews checklist (PRISMA-ScR; S1 PRISMA Checklist) [20–22].

### Eligibility criteria

Publications were included if they utilized the term “direct to consumer” to refer to a health-related product or service and also discussed ethics related to DTC healthcare. Publications were excluded if they: (a) focused solely on DTC *advertising* of a traditional product or service; (b) made no mention of ethics in the main text (i.e., some included a statement that their study had undergone ethics review but no other discussion of ethics); (c) did not mention the term “direct to consumer” to refer to a health-related product or service in the main text (i.e., it came up in references only); or (d) the publication focused primarily on DTC genetic testing.

### Search strategy

**Fig 1:**
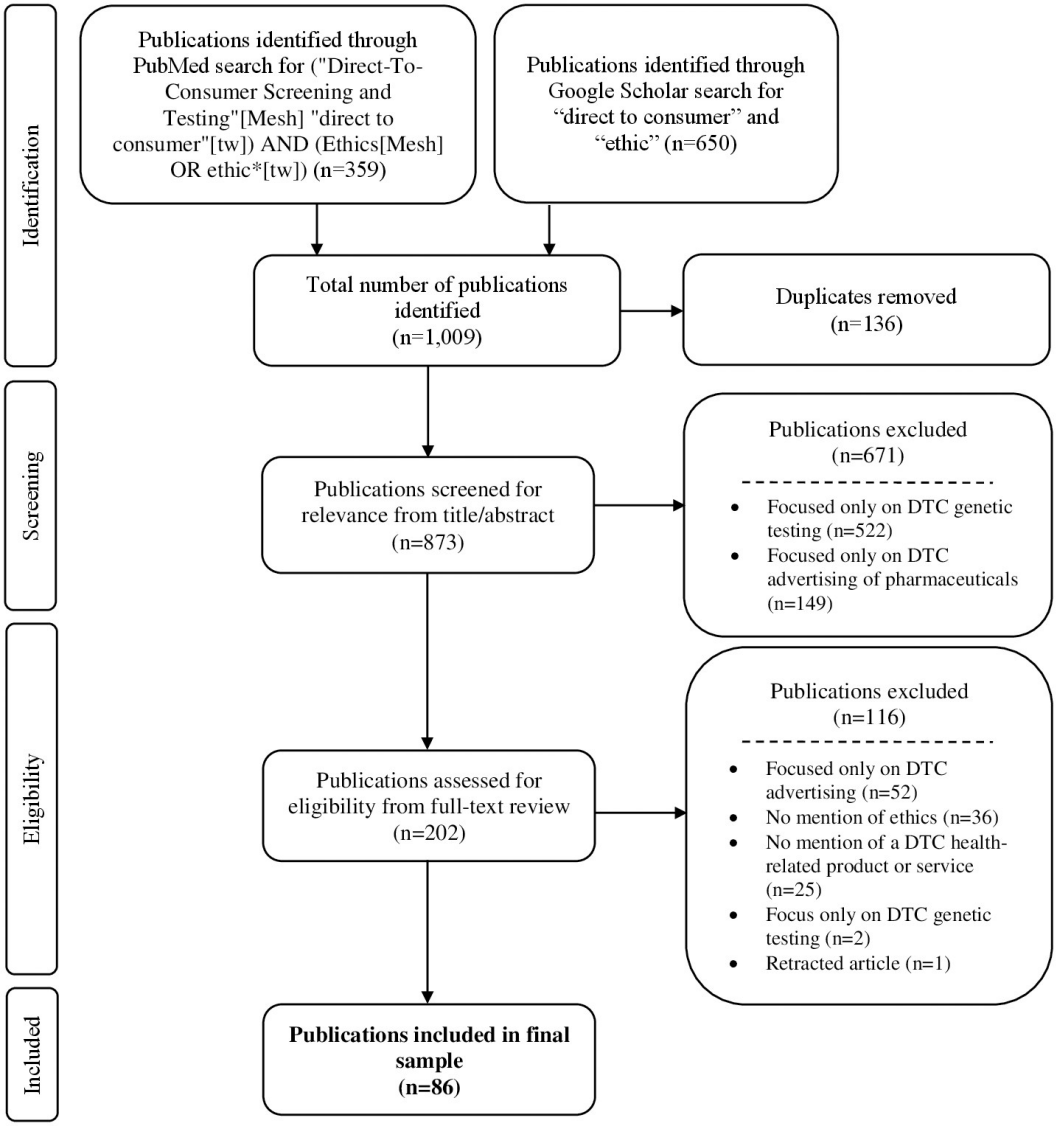
PRISMA flowchart depicting the process of identifying and screening publications included in the scoping review.

We searched PubMed and Google Scholar in December 2021 using keywords related to DTC healthcare and ethics as shown in the PRISMA (Fig 1). We restricted our search to English-language publications and book chapters published within the past ten years. For the Google Scholar search, we reviewed the first 650 results as relevancy declined after 450 results.

### Selection of sources of evidence

All resulting publications (n = 1,009) were imported into Covidence, an online systematic review tool. Covidence automatically identified duplicates (n = 136) for removal; these were reviewed by one author (AN) to ensure accuracy.

Next, one author (AN) screened the title and abstract of each publication and excluded those primarily focused on: (a) DTC genetic testing, as there is extensive literature on this topic with multiple scoping reviews [10–12]; and (b) DTC advertising of pharmaceuticals, as they did not represent a novel DTC product or service. A second coder (NW) screened 10% of the publications to ensure reliability; IRR was 94.3%. Any publication for which the focus was unclear was included in the next stage of review.

The full text of all remaining publications (n = 202) was reviewed for relevance by one author (AN). All publications for which there was uncertainty were reviewed and discussed by three authors (AN, LK, AW) until consensus was reached.

### Data extraction and analysis

We extracted data for salient characteristics of each publication, such as the primary DTC product or service of interest, type of study (i.e., empirical, conceptual, or legal), year of publication, and geographical context. Next, all authors reviewed a subset of included publications in order to develop a codebook that consisted of ethical issues related to DTC healthcare (S1 Codebook). All codes were created inductively, based upon reviewing the data rather than utilizing a set of preidentified codes. In determining what constituted an “ethical issue,” we found that publications differed in their level of depth of ethical discussion: most pointed to a problematic practice (e.g., insufficient information provided to consumers) but varied in whether they explicitly connected it back to an ethical requirement (e.g., informed consent) or an ethical principle (e.g., autonomy). We also found that most concerns raised about DTC health products were implicitly related to ethics, regardless of whether an explicit connection was made. For example, lack of adequate regulation, while not a principle-based ethical issue in itself, was frequently raised as a proxy for the discussion of harm to consumers. Because the papers themselves took a broad view of ethical concerns and more frequently pointed to practices rather than underlying principles, we adopted a similarly expansive view in our determination of ethical issues in the interest of capturing the broad range of issues discussed in the literature.

To ensure the reliability of our codebook, two coders (AN and LK) piloted our codebook in two preliminary rounds. First, five publications were coded by both coders, and discussions were held to resolve any disagreements and clarify the codes. Next, ten publications were coded by both authors, and discussions were held to resolve any disagreements. Subsequently, one author (AN) coded each publication in Dedoose and brought all questions regarding code application for group discussion (among the three authors) to reach consensus.

## Results

### Characteristics of included publications

**Fig 2:**
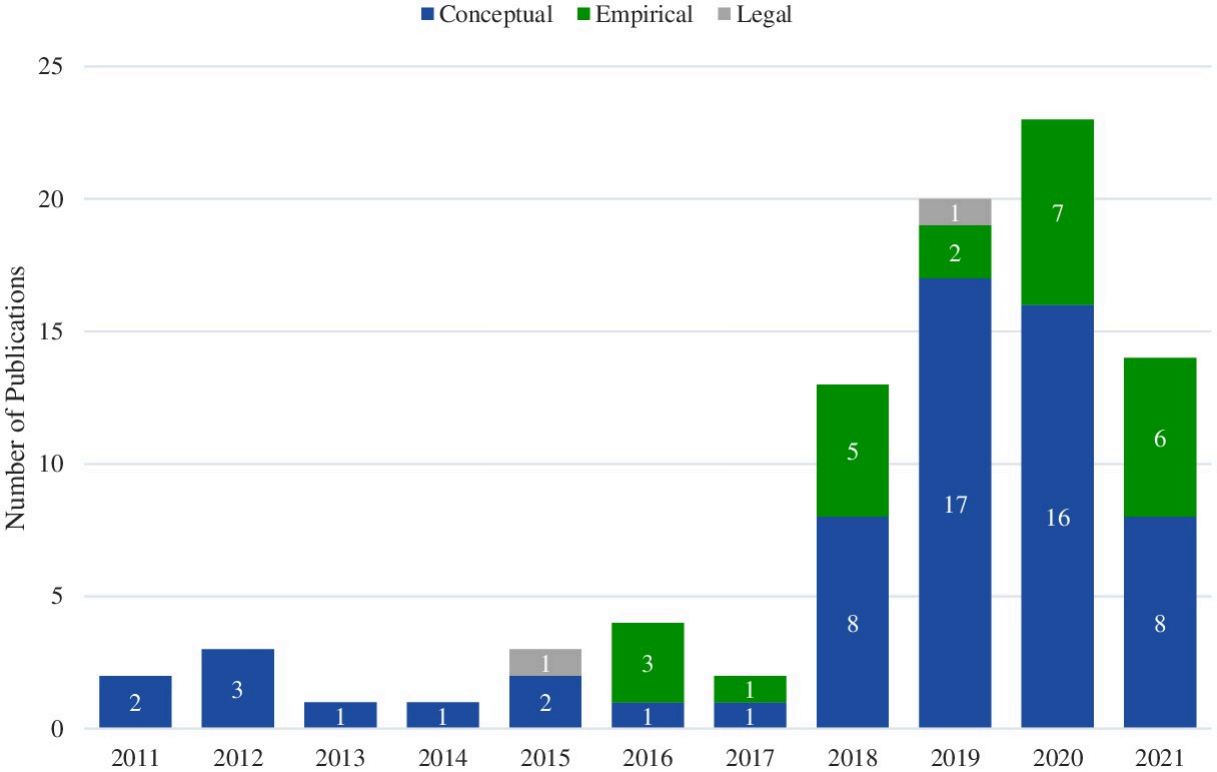
Publications discussing the ethics of DTC healthcare products and services (N = 86) displayed by publication year and article type (i.e., conceptual, empirical, legal).

We identified 86 English-language publications discussing the ethics of DTC healthcare products or services published between January 2011 and December 2021 (Fig 1). The majority (81%; n = 70) were published between 2018 and 2021, and most (70%; n = 60) were conceptual in nature (Fig 2). Approximately half (49%; n = 42) did not specify a particular geographical context; 38% (n = 33) focused exclusively on the U.S. context and 13% (n = 11) centered on or included significant mention of locations outside the U.S. Some of these focused exclusively on a single context (e.g., a study of Canadian healthcare providers offering DTC stem cell procedures), whereas others discussed international contexts in the setting of regulatory discussions (e.g., European laws pertaining to data collected from DTC neurotechnology devices). Most publications that included a significant mention of a non-U.S. context centered on DTC stem cell clinics (n = 6) or DTC neurotechnology devices (n = 3).

**Fig 3:**
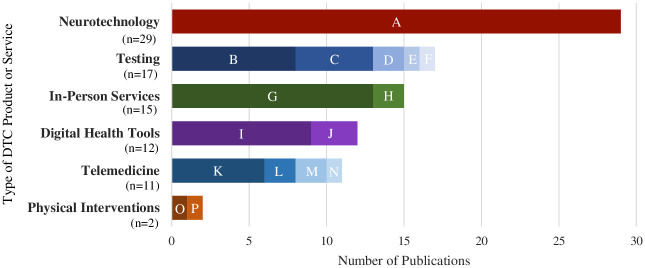
Types of DTC products and services mentioned by publications (N = 86) in our sample. Subcategorizations include: (A) Noninvasive neural & neurostimulation devices (n = 29); (B) Laboratory testing (n = 8); (C) Disease screening (n = 5); (D) COVID-19 testing (n = 2); (E) Fertility testing (n = 1); (F) Alzheimer’s disease testing (n = 1); (G) Stem cells clinics (n = 13); (H) Medical imaging (n = 2); (I) Wearables and general health apps (n = 9); (J) Mental health apps (n = 3); (K) Dermatology (n = 6); (L) Psychotherapy (n = 2); (M) Prescription medications (n = 2); (N) Allergies (n = 1); (O) Orthodontics (n = 1); (P) Audiology (n = 1).

We categorized publications about DTC healthcare products and services into six types (Fig 3). *Neurotechnology* publications centered on noninvasive neural recording and neurostimulation products, such as electroencephalography (EEG) and transcranial direct current stimulation (tDCS) devices. Publications focused on *testing* discussed laboratory tests such as sexually transmitted infections tests and disease screening (e.g., for atrial fibrillation). Publications about *in-person services* primarily centered on walk-in stem cell clinics, but also included medical imaging services such as DTC fetal ultrasounds. Publications categorized as *digital health tools* predominantly discussed wearables and apps, and most publications about *telemedicine* focused on dermatology. Within *physical interventions*, one publication discussed DTC teeth aligners, and another centered on DTC hearing aids.

Neurotechnology-related publications were most prevalent, likely due to multiple commentaries accompanying a relevant journal article (n = 13) [23] and chapters (n = 9) in a neurotechnology book [24]. Other publications from special issues included five related to dermatology and telemedicine [25–29] and four related to DTC lab testing [7,30–32].

**Fig 4:**
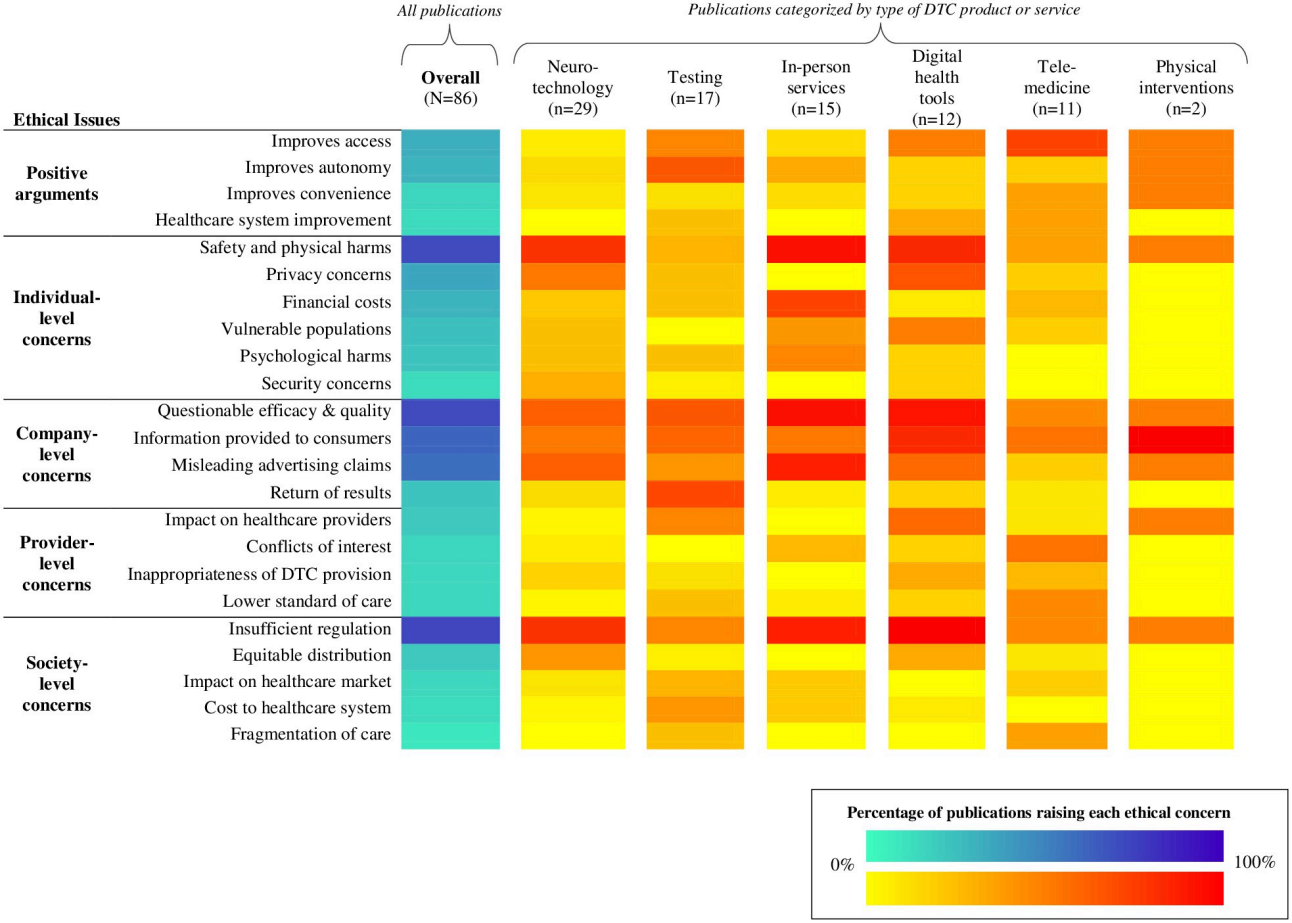
Left (blue heatmap): frequency of specific ethical issues across all publications in our sample, with dark blue indicating the most commonly discussed issues. Right (red heatmaps): frequency of ethical issues within each type of DTC product or service, with dark red indicating the most frequently discussed issues.

A myriad of ethical issues were raised across all publications in our sample, with ethical concerns outnumbering ethical arguments in favor of DTC healthcare. The frequency of specific ethical issues raised by *all* publications is depicted in Fig 4 (left; blue heatmap) and elaborated upon below. Ethical concerns that appeared most frequently are organized by primary stakeholder to which the ethical issue was relevant: individual consumers, companies selling DTC healthcare products or services, healthcare providers, and society writ large. Given that many ethical concerns have relevance for multiple stakeholders (i.e., *individuals* may be harmed by misleading claims, but it is *companies’* responsibilities to make truthful claims), our characterization is a general conceptualization to aid discussion and analysis.

Fig 4 (right; red heatmaps) illustrates the frequency of ethical issues *within* each type of DTC product or service. Positive ethical arguments in favor of DTC healthcare are described prior to the review of ethical concerns.

### Ethical arguments in favor of DTC healthcare

#### Improves access

Nearly a third of all publications (31%; n = 27) discussed how DTC healthcare may help individuals overcome various barriers to accessing healthcare. Potential reductions in cost were noted by publications about DTC teledermatology [28], DTC stem cells [33], DTC testing [7,34,35], and DTC hearing aids [36]. It was argued that mental health apps and virtual therapy extend access to those in remote areas or to those unable to leave their home for physical or psychological reasons [23,37]. The DTC approach also allows individuals to communicate with their therapists anytime and from anywhere, facilitating continuous support [37]. Increased accessibility was also noted in the context of DTC telemedicine [38–40], where it can provide an alternative to emergency room visits, particularly in rural locations [38]. Arguments were made that DTC telemedicine expands access to providers, such as psychologists [8,37], and to those seeking wellness, education, peer counseling, and referral services [41].

#### Improves autonomy

Several publications (29%; n = 25) noted that DTC healthcare has created opportunities for individuals to take charge of their own healthcare. For example, in the realm of DTC testing, it was argued that individuals have greater control over their personal health information, including what tests they order and who they share data with [7,30–32,34,35,42]. Regarding DTC tele-mental health platforms, one article emphasized that they allow greater consumer choice as individuals can select their provider from an array of specialists [37]. Furthermore, in the realm of digital health tools, it was argued that more informed patients can be more actively involved in their care and treatment decisions [43].

#### Improves convenience

In addition to offering increased access, some publications (16%; n = 14) pointed out the increased convenience afforded by DTC healthcare. DTC orthodontic aligners, for example, eliminate the need for orthodontist appointments as the product is ordered online and shipped to individuals’ homes [44]. Convenience in terms of reducing appointments was also discussed in relation to DTC telemedicine and testing [35,38,39], and publications about DTC tele-mental health services noted that they allow individuals the flexibility to easily select the meeting time and duration of their services [8,37].

#### Healthcare system improvement

Some publications (14%; n = 12) argued that DTC healthcare improves the traditional healthcare system by improving workflow and efficiency. For example, it was argued that digital health apps could reduce costs and lead to better overall health outcomes [45]. Arguments were also made that DTC testing could improve the healthcare system by allowing consumers to screen themselves, which would allow them to avoid doctor’s office visits, especially when the results are normal [7].

#### Other positive arguments

Other arguments made in favor of DTC healthcare included increased options for those wanting to try non-standard therapies [33,46], anonymity for those who might not seek healthcare services otherwise [25,37], and early diagnosis [35].

### Individual-related ethical concerns

#### Safety and physical harms

Safety and physical harms were the most frequently discussed individual-related ethical issue in our sample (66%; n = 57). Many publications expressed concerns about the lack of sufficient evidence of safety and/or rigorous testing for DTC healthcare products and services [46–51]. This was particularly salient for articles about unapproved stem cell procedures, which discussed the risk of serious adverse events such as lesions and blindness [48,49,52,53]. Publications about neurotechnology frequently touched upon safety concerns related to short- and long-term effects of neurostimulation [9,23,54–62], with some mentioning the possibility of minor to moderate adverse events with improper use, such as skin rashes and burns [54,55]. Worries of physical harms were also raised with DTC teeth aligners regarding the possibility of adverse oral health outcomes [44], and with DTC imaging tests regarding the risk of radiation exposure [34].

#### Privacy

Approximately one-third of publications (34%; n = 29) mentioned privacy concerns, particularly in relation to neurotechnology, digital health tools, and testing, as these products involve some form of data collection. Neurotechnology-related concerns focused on the privacy and integrity of personal and brain data [2,56,59,63–65] and the potential sharing of this data with third parties [23]. Privacy issues related to digital health tools included location tracking for wearable devices [66], insufficient protection of data for mental health apps [67], and overly complex terms and conditions [47]. Testing-related privacy worries encompassed the potential for compromised consumer data, including health and financial information [68]; insufficient privacy considerations in the informed consent process for DTC laboratory testing [35]; and concerns about biomarker-based discrimination [69].

#### Financial costs

Nearly a third of publications (29%; n = 25) mentioned financial costs—and in particular the out-of-pocket expenses—associated with DTC products and services. Many highlighted the significant financial investment required to obtain DTC stem cell treatments [49,70–72], which can cost tens of thousands of dollars [46,53,73]. Concerns were also raised about the potential financial burden of DTC testing such as cardiac screening and p-tau blood testing [69,74]. In addition, some publications noted how DTC telemedicine costs may not be covered by insurance, leading to out-of-pocket expenses [25,39], while others highlighted the cost of consumer neurotechnology devices [75,76].

#### Vulnerable populations

Concerns were raised in approximately a quarter of publications (24%; n = 21) about the exploitation of vulnerable populations by purveyors of DTC products and services. Stem cell clinics were a specific focus of concern, particularly as discussed in the context of parents who opt for regenerative treatments for their children [46,48,49,51,52,72]. A number of publications expressed concerns about the use of DTC therapy or mental health apps by children or those with mental health conditions, as these vulnerable populations may be more susceptible to exploitation [8,67,77–79]. These publications called for extra care to be taken with vulnerable populations regarding informed consent [79], user authentication [8], and data protection [78]. Furthermore, some publications argued against the use of neurostimulation devices in children [60,80].

#### Psychological harms

The psychological harms of DTC products and services were discussed in 23% (n = 20) of publications. Topics included the harms associated with disappointment from expectations about the success of a service [52,65], anxieties resulting from false positives related to testing and imaging [75,81,82], and the psychological impact of receiving upsetting or unclear information [59].

#### Security

Some publications (14%; n = 12) raised concerns about security, emphasizing the potential harm to consumers from inadequate data protection measures. Most concerns were neurotechnology-related, with publications questioning the security of brain-related data stored on smartphones [65] or processed in the cloud [83]. Others discussed varying cybersecurity risks associated with different types of neurotechnologies [23], suggesting that neuromodulation products may pose fewer concerns than neuromonitoring devices, which collect brain data [59]. Non-neurotechnology-related security issues involved inadequate security for mobile health apps [67] and wearable devices [66].

#### Other concerns

Other individual-related issues included the ability of consumers to make informed risk-benefit judgments in the DTC context [47], opportunity costs associated with choosing DTC options over traditional healthcare alternatives [74], and harms stemming from the use of neurological data to establish culpability [63].

### Company-related ethical concerns

#### Questionable efficacy and quality

The majority of publications (70%; n = 60) discussed concerns regarding the efficacy and quality of DTC products and services. Numerous publications related to stem cells highlighted their reliance on limited [48,73] or “ambiguous” evidence [84]. Other criticisms focused on experimental techniques in neurotechnology [55,85–87] and digital health tools, which may not be rigorously tested [47] or have strong supporting evidence [8,41,45]. Articles critiqued the quality of DTC lab tests [30,31] as well as the lack of clinical evidence and consensus supporting their widespread use [74,81,88].

#### Insufficiency of information provided to consumers

Many publications (59%; n = 51) emphasized the importance of individuals having accurate and complete information to make informed choices [44,47]. In the context of DTC testing, articles noted that individuals should be informed of appropriate indications for testing [89], the possibility of incidental findings [34], and factors affecting laboratory results [30]. Stem cell clinics were criticized for not providing sufficient information to individuals about the lack of medical evidence for the safety and efficacy of their treatments [46,49,70]. Among neurotechnology publications, concerns were raised regarding the disclosure of potential adverse effects [57,90,91] and the experimental nature of neurostimulation [55], as well as for obtaining informed consent for the collection of brain data [83]. Some questioned whether purveyors of DTC healthcare should be held to medicolegal standards of informed consent [92]. Others noted that with consumer health apps, individuals often proceed through the informed consent by clicking through without carefully reading the text-laden forms [67,79], raising concerns about individuals’ awareness of the data being collected [8,66,87,93]. Similar concerns about terms of service agreements were raised with tele-mental health services [78].

#### Misleading advertising

A considerable number of publications (56%; n = 48) across all types of DTC products and services expressed concerns about misleading marketing. In-clinic commercial therapies such as stem cell therapies were criticized for making unsubstantiated claims of treatment efficaciousness [48,50,53], misleadingly marketing their interventions as safe [46], and inaccurately making claims about regulatory compliance [52]. Similarly, publications discussing digital health tools noted that companies’ claims may be based on questionable evidence [45] and may misrepresent consumer liability [68]. In the realm of DTC neurotechnology, critiques were raised regarding companies’ misrepresentation of product capabilities [56], with some noting a shift to wellness claims to avoid classification as medical devices [85,86].

#### Return of results

Approximately a quarter of publications (23%; n = 20) expressed concerns about information provided to consumers after using DTC products and services. These included worries about individuals misinterpreting test results [1,31,94] or having difficulty understanding results without medical guidance [30,35]. Relatedly, there were concerns about “overinterpretation” and false reassurance from false negative results [30,74,95], as well as the possibility of an incorrect diagnosis and increased healthcare costs [7]. Moreover, some articles raised questions about whether DTC testing provides actionable information [31] or if unclear or incomprehensive results could lead to unnecessary follow-up [30]. Regarding neurotechnology, worries were expressed about whether and how companies should communicate incidental findings (e.g., brainwave patterns that may be indicative of epilepsy or sleep disturbances) to users [54].

#### Other concerns

Other company-related ethical issues included lack of standardization [27,94], unclear accountability for companies [78,79], and questionable utility of DTC tests [74].

### Healthcare provider-related ethical concerns

#### Impact on healthcare providers

Some publications (21%; n = 18) discussed the implications of DTC products and services for healthcare professionals. It was argued that DTC healthcare might lead individuals to bypass doctors and their qualified medical guidance [18,39], thereby posing safety risks [74] and jeopardizing the therapeutic relationship [89,94]. There were concerns that consumers might not discuss their DTC test results or prescriptions with their primary healthcare providers [35,39], instead relying on DTC technology [47]. Additionally, individuals might distrust providers who offer different recommendations or diagnoses than their DTC devices [43]. As a result, articles highlighted the need for providers to familiarize themselves with DTC healthcare as they may increasingly be required to advise patients on digital health products [18], explain the evidence and utility of DTC healthcare to patients [43,68,77], or manage patients’ questions after using DTC healthcare [18,96].

#### Conflicts of interest

Concerns about conflict of interest (COI) were raised about healthcare providers who work for DTC companies (16%; n = 14); these worries arose most frequently in the context of DTC telemedicine. Arguments were made that providers have a financial COI with DTC prescription platforms since prescribing medications that increase the company’s profits is essential for the platform’s survival [25,26,97]. The issue of COI was also raised with stem cell therapy clinics [53], providers of neurostimulation [55], telemedicine [39], and digital health tools [68] who all have a financial interest in profit maximization.

#### Inappropriateness of DTC provision model

Several publications (16%; n = 14) raised concerns about the suitability of certain services being provided through a DTC provision model. This was primarily discussed in relation to DTC telemedicine and digital health tools. For example, it was noted that DTC telemedicine services for erectile dysfunction (ED) might be inappropriate given that the standard of care for ED screening is a physical examination [38,97]. It was also argued that DTC mental health apps may not be appropriate for those with severe mental disorders [8,93].

#### Lower standard of care in the DTC context

Some publications noted how the standard of care differs in DTC healthcare as compared to traditional healthcare (15%; n = 13). For example, in traditional settings, patients typically have continuity of care with the same provider, but in the DTC context, there is no guarantee that individuals will be seen by the same provider in subsequent visits [27]. Furthermore, concerns were raised regarding the provision of DTC products and services in the absence of proper medical histories, informed consent, and validation of patients’ identities [25,39,40,68]. For example, in teledermatology, it was argued that lack of a detailed medical history can lead to the prescription of contraindicated medications [25]. Additionally, in a DTC telemedicine context, there is a lack of “door-handle” questions [97], that is, extra questions asked by the patient that are unrelated to the main goal of the visit.

#### Other concerns

Other healthcare-related ethical issues include variability in provider training and licensing [25,38,40] and lack of awareness among healthcare providers of the regulatory status of the products and services they are offering [55].

### Society-related ethical concerns

#### Insufficient regulation

Most publications (72%; n = 62) critiqued the regulation of DTC healthcare. Arguments were made that regulatory gaps and loopholes, particularly around neurotechnology devices and digital health tools marketed for wellness purposes, allow companies to market their products with insufficient safety and efficacy data [8,43,57,80,98,99]. In the context of DTC stem cell clinics, publications argued that regulation was ineffective and weak, with piecemeal and non-systematic regulatory action that has allowed the market for unapproved stem cell products to flourish [46,48,50,52]. In the realm of digital health, scholars pointed to a mismatch between the current regulatory framework and the evolving digital health environment [8,68,77,93], pointing out how the sheer number of digital health products and the fast pace at which they can be produced present challenges for FDA regulation [93]. In addition, FDA regulation largely focuses on the risk of physical adverse events, whereas digital health products may present harms to mental health or raise issues related to informed consent and privacy [8,93]. Regarding DTC lab testing, articles pointed to the absence of HIPAA protections [69] and the lack of established protocols for returning incidental or secondary findings [34].

#### Equitable distribution

Concerns about the fair distribution of resources in society were predominantly raised in relation to neurotechnology and digital health tools (21%; n = 18). Some noted that DTC platforms are not designed for underserved populations, given the limited availability of non-English-language options [47] or acceptance of Medicaid [28]. Others argued that expensive cognitive enhancement neurotechnologies and digital health tools might exacerbate current gaps in inequality [23,47,55,58,59,93]. However, it was noted in at least one publication that just distribution is only an ethical concern if a given product indeed confers a benefit or advantage [56]. Assuming that a DTC product or service does provide benefit, arguments were made that they should be widely accessible [23] and benefit the underserved [80].

#### Changes to the healthcare market

Several publications discussed how DTC products and services will impact the healthcare market (15%; n = 13). For example, labs may need to adapt to meet the new demand [95] and offer lab testing consultation services [94]. Regarding DTC telemedicine, concerns were raised that the rapid growth of companies might outpace the ability of healthcare stakeholders, such as payers and providers, to effectively respond and adapt [38]. Also, it was argued that DTC telemedicine companies would enable more prescriptions to be written outside the traditional therapeutic relationship [39].

#### Cost to the healthcare system

Several publications addressed the potential cost implications of DTC healthcare (14%; n = 12). Specifically, concerns were raised that false positives in the context of DTC testing and screening could lead to unnecessary follow-up services [30,74,82,89], forcing the healthcare system and insurers to incur additional costs [7,31,69,92]. A related concern was that physicians might be compelled to practice “defensive medicine” and order additional low-value tests or treatments as a precautionary measure to avoid potential legal repercussions [43].

#### Fragmentation of care

Some publications (9%; n = 8) discussed concerns about the potential fragmentation of care resulting from DTC products and services. Scholars pointed out that DTC telemedicine services are not typically integrated with individuals’ existing medical records [38], and with DTC testing, individuals are responsible for consolidating their data into a universal health record [31,32]. Articles also highlighted the lack of care coordination between DTC testing or telemedicine services and individuals’ primary care physicians [25,39,40], placing the burden on individuals to determine the need for follow-up care [89].

#### Other concerns

Additional ethical concerns at the societal level included the medicalization of normal states leading to overdiagnosis [87], blurred boundaries between treatment and lifestyle [18,87], and the role of algorithmic bias in exacerbating health disparities [47,59,83].

## Discussion

Our findings revealed a wide range of ethical issues—both positive and critical—in the academic literature regarding DTC healthcare products and services. Positive arguments centered on how DTC products and services provide consumers with increased access, convenience, and autonomy. The most frequently expressed ethical concerns related to insufficient regulation, as well as questionable efficacy and quality—reflecting a growing unease with oversight of novel DTC provision models that upend traditional pathways of healthcare delivery. This sentiment arose in other ethical concerns, such as those related to misleading marketing claims, the inappropriateness of DTC healthcare, and its comparatively lower standard of care.

The potential physical harm to consumers emerged as the third most frequently raised ethical concern, particularly in discussions of neurotechnology devices [9,23,54–62], stem cell clinics [48,49,52,53], telemedicine prescriptions [25], and physical interventions like DTC teeth aligners [44]. This concern was less prevalent in publications discussing the DTC provision of health information, such as that provided by laboratory tests, neurorecording devices, or imaging services. For those services, privacy was a frequently raised concern [35,68,69], due to the lack of existing laws protecting sensitive health information.

Given that DTC healthcare primarily involves a transaction between an individual consumer and a company, it is not surprising that many ethical issues centered on the dynamics of the consumer-company relationship, such as those related to financial cost and vulnerable populations. Other salient areas of focus surrounded information provided to the consumer prior to the transaction—such as in the informed consent [67,79,83,92]—as well as information returned to the consumer, with arguments highlighting the potential for consumers to misinterpret their results [1,14,31].

Beyond the consumer-company relationship, scholars pointed to the implications that DTC healthcare may have on various stakeholders, including healthcare providers, the healthcare system, and society writ large. It was argued that even healthcare providers who have no relationship with DTC companies may feel its effects, in terms of needing to familiarize themselves with DTC products and services to field questions from patients [18,43,68,77,96]. Societal-level ethical issues presented contrasting viewpoints. While some advocated for its potential to reduce costs [28], others expressed concerns about increased system costs [7,31,69,92]. Likewise, opinions differed on whether DTC products effectively increase access for underserved populations [38] or fail to reach them [28,47].

While our study did not include articles addressing the ethics of DTC genetic testing, as there are multiple reviews on the subject [10–12,100], a comparison of findings reveals several shared themes. Commonalities primarily revolved around concerns related to information provided to consumers, privacy, and potential misinterpretation of results. However, there are notable differences. The DTC genetic testing literature places more emphasis on psychological harms (e.g., unnecessary stress) stemming from test results, while (indirect) physical harms are discussed to a lesser extent, as genetic tests provide information and not interventions. In addition, while concerns relating to potential conflicts of interest have been discussed in the DTC genetic testing literature [101], they seem to be less prominent, possibly because the integration of physicians in the provision of DTC genetic testing has varied over the years and across different companies [102].

Assessing the occurrence of ethical issues across different types of DTC products or services can provide valuable insights. First, it can help identify ethical concerns that may have been overlooked within specific medical domains. For example, although concerns about fragmented care were raised in relation to telemedicine and DTC testing, it may be an issue worthy of consideration for other digital health tools. Second, charting ethical issues across types of DTC healthcare may yield a roadmap for ethical issues that are likely to arise in the future for emerging types of DTC products and services. Third, our findings demonstrate significant overlap regarding the ethical issues identified across types of DTC products and services, highlighting the importance of understanding DTC healthcare as a broad social phenomenon.

### Limitations

Our study has several limitations. First, it was limited to English-language publications utilizing the terms “direct-to-consumer” and “ethics,” thus publications discussing ethical issues related to DTC healthcare without explicitly using these terms were not included in our sample. Second, our literature search was limited to two databases, PubMed and Google Scholar, thus we may have missed pertinent studies indexed in other databases. Third, we relied on authors’ labeling of products as DTC, rather than independently assessing whether they met certain criteria. Fourth, we did not code for the depth of discussion of ethical issues, thus our results do not distinguish between brief references to ethical issues and more comprehensive analyses.

## Conclusions

In sum, this scoping review offers insights into the ethical issues raised across a wide variety of literature about DTC healthcare products and services. Our findings shed light on the cross-cutting nature of the phenomenon and underscore the need to develop novel ethical frameworks tailored to the unique stakeholders and dynamics introduced by DTC models. Future research should focus on understanding whether the ethical issues raised by publications in our sample are supported by empirical evidence, as well as examining how specific medical domains have navigated the challenges of DTC healthcare.

## Supporting information

S1 PRISMA Checklist Preferred Reporting Items for Systematic Reviews and Meta-Analyses extension for Scoping Reviews (PRISMA-ScR) Checklist. (PDF)

S1 Codebook Data analysis codebook. (PDF)

S1 Data Repository Extracted data. (XLSX)
