# Supplementary material for: Ethical issues in direct-to-consumer healthcare: A scoping review
Source: PLOS Digit Health. 2024 Feb 13;3(2):e0000452. doi: 10.1371/journal.pdig.0000452 (PMC10863864; doi:10.1371/journal.pdig.0000452)
Supplement: S1 Codebook — (PDF) [file pdig.0000452.s002.pdf]

S1 Appendix. DTC-ethics scoping review codebook.

0. **Geographical location *(required code)***
   - 0.1 U.S.-Only
   - 0.2 Outside U.S.
   - 0.3 General
1. **Type of research *(required code)***
   - 1.1 Empirical
   - 1.2 Conceptual
   - 1.3 Legal analysis
2. **Year of publication *(required code)***
   - 2.1 2011
   - 2.2 2012
   - 2.3 2013
   - 2.4 2014
   - 2.5 2015
   - 2.6 2016
   - 2.7 2017
   - 2.8 2018
   - 2.9 2019
   - 2.10 2020
   - 2.11 2021
3. **Modality *(required code)***
   - 3.1 In-clinic/walk-in service
   - 3.2 Physical intervention
   - 3.3 Telemedicine
   - 3.4 Testing (home + lab)
   - 3.5 Neurotechnology
   - 3.6 Digital health tools
4. **Positive arguments in support of DTC**

   4.1 Improves access

   Mentions how DTC services/products can help individuals overcome financial, geographical, and social barriers

   4.2 Improves convenience

   Mentions how DTC improves convenience (this is implicitly the same ethical issue as access, but often discussed separately)

   4.3 Improves autonomy

   Mentions how consumers of DTC products are better informed about their treatment options, are directing their own care, and empowered to obtain care on their own terms

   4.4 Healthcare system improvement

   Mentions of how DTC healthcare can improve system workflow/efficiency, thereby leading to improved use of resources and better health outcomes
5. **Company-level concerns**

   5.1 Questionable efficacy and quality

   Questionable efficacy or quality of a product

   5.2 Misleading advertising claims

   The use of misleading, fraudulent, or hyperbolic marketing claims; the promising benefits from experimental/unproven services; or lack of transparency about what the product can/cannot do

   5.3 Insufficiency of information provided to consumers

   Issues related to consumers having sufficient information to make an informed choice prior to obtaining a DTC product or service, such as information regarding potential risks and benefits and how data will be collected or used

   5.4 Return of results

   Appropriate communication of results, information, and incidental findings, as well consumer understanding of these results, such as overinterpretation or misinterpretation, as well as lack of sufficient information or health literacy to accurately interpret results

5.5 Other

6. **Individual-level concerns**

6.1 Safety/physical harms
Service/product potentially leads to some physical harm to the consumer
6.2 Non-physical/psychological harms
Psychological harms to consumers through use of service/product
6.3 Financial costs
The cost of service/product is expensive to the consumer, typically out-of-pocket
6.4 Vulnerable populations
Notes added risks if service/product is used by children or other vulnerable individuals
6.5 Privacy
Discusses adequate protection of health data, data sharing, HIPAA, or risks of data identifiability
6.6 Security
Concerns related to data security (differs from privacy in that the focus is on protection from potential security breaches, though ultimately raises similar underlying ethical concerns regarding unauthorized data sharing)
6.7 Other

7. **Provider-level concerns**

7.1 Lower standard of care
Changes to standard of care in the DTC setting, such as lack of "door-handle" questions
7.2 Appropriateness
Mention of needing to determine appropriateness of certain services in DTC health business models
7.3 Conflict of interest
Mention of how physicians hired by DTC companies may face a financial conflict of interest that may impact patient care
7.4 Impact on healthcare providers
Mentions how the traditional physician-patient relationship may change as a result of consumers using DTC services, such as consumers not informing their primary care providers of their use of DTC services
7.5 Other

8. **Societal-level concerns**

8.1 Impact on healthcare market
Mention of how DTC healthcare products and services can disrupt the traditional healthcare market
8.2 Cost to healthcare system
Mention of how services can lead to unnecessary testing which increases costs to the system, uses of scare resources, or is wasteful
8.3 Fragmentation of care
Mention of how DTC services fragment traditional health care delivery or how they are point solutions and/or create "siloes" rather than holistic approaches
8.4 Equitable distribution/distributive justice
Mentions of how some people will be excluded from using DTC services/products because of access barriers, such as cost, language, and technology
8.5 Insufficient regulation
Mentions how DTC products and services are not adequately regulated which exacerbates ethical concerns and can cause downstream harm to consumers
8.6 Other
